# The betrayed thief – the extraordinary strategy of *Aristolochia rotunda* to deceive its pollinators

**DOI:** 10.1111/nph.13210

**Published:** 2014-12-08

**Authors:** Birgit Oelschlägel, Matthias Nuss, Michael von Tschirnhaus, Claudia Pätzold, Christoph Neinhuis, Stefan Dötterl, Stefan Wanke

**Affiliations:** 1 Institut für Botanik, Technische Universität DresdenZellescher Weg 20b, 01062, Dresden, Germany; 2 Senckenberg Naturhistorische Sammlungen Dresden & Museum für TierkundeKönigsbrücker Landstraße 159, 01109, Dresden, Germany; 3 Fakultät Biologie, Universität BielefeldPostfach 100 131, 33501, Bielefeld, Germany; 4 Lehrstuhl für Pflanzensystematik, Universität BayreuthUniversitätsstraße 30, 95447, Bayreuth, Germany

**Keywords:** *Aristolochia*, chemical mimicry, Chloropidae, Croatia, deception, kleptoparasites, pollination system

## Abstract

- Pollination of several angiosperms is based on deceit. In such systems, the flowers advertise a reward that ultimately is not provided. We report on a previously unknown pollination/mimicry system discovered in deceptive *Aristolochia rotunda* (Aristolochiaceae).
- Pollinators were collected in the natural habitat and identified. Flower scent and the volatiles of insects (models) potentially mimicked were analyzed by chemical analytical techniques. Electrophysiological and behavioral tests on the pollinators identified the components that mediate the plant–pollinator interaction and revealed the model of the mimicry system.
- The main pollinators of *A. rotunda* were female Chloropidae. They are food thieves that feed on secretions of true bugs (Miridae) while these are eaten by arthropod predators. Freshly killed mirids and *Aristolochia* flowers released the same scent components that chloropids use to find their food sources. *Aristolochia* exploits these components to deceive their chloropid pollinators.
- *Aristolochia* and other trap flowers were believed to lure saprophilous flies and mimic brood sites of pollinators. We demonstrate for *A. rotunda,* and hypothesize for other deceptive angiosperms, the evolution of a different, kleptomyiophilous pollination strategy. It involves scent mimicry and the exploitation of kleptoparasitic flies as pollinators. Our findings suggest a reconsideration of plants assumed to show sapromyiophilous pollination.

## Introduction

Angiosperms are the most successful terrestrial plants and predominantly pollinated by animals, especially insects (Faegri & van der Pijl, 1979; Ollerton *et al*., 2011). Floral adaptations that increase pollen transfer efficiency and foster cross-pollination have indisputably been a driving force of angiosperm evolution and diversification (Hu *et al*., 2008; Kay & Sargent, 2009; Peakall *et al*., 2010). Typically, angiosperms share mutualistic interactions with their pollinators, but in an estimated 4–6% of flowering plants, pollination is based on deceit (Renner, 2006), whereby plants advertise a reward but ultimately do not provide it. In such systems, only the plant benefits, because pollinators are unable to recognize the fake reward or may not be able to distinguish between rewarding and nonrewarding flowers. Deceptive systems range from imitation of mating partners to imitation of food sources, or sites for oviposition by structural, visual, or scent signals (Dafni, 1984; Schiestl *et al*., 1999; Stensmyr *et al*., 2002; Brodmann *et al*., 2009; Stökl *et al*., 2010). Although deceptive pollination systems have often been postulated, decipherment of all partners and the cues mediating the interaction are limited to few systems (e.g. Stensmyr *et al*., 2002; Schiestl *et al*., 2003; Brodmann *et al*., 2008).

*Aristolochia* represents one of the most fascinating plant genera and is well known for its peculiar, proterogynous (stigma is receptive before pollen is released) flowers specialized for the trapping, retention, and release of pollinators (Correns, 1891; Oelschlägel *et al*., 2009). According to current knowledge, virtually all species cheat pollinators. Some species produce flowers that are among the largest on Earth (Bello *et al*., 2006). Although *Aristolochia* is distributed nearly worldwide from temperate to tropical habitats and morphologically adapted to different climatic zones, the basic flower layout is homogeneous (González & Stevenson, 2000). *Aristolochia* is pollinated by flies (Diptera), such as Calliphoridae, Ceratopogonidae, Drosophilidae, Mycetophilidae, and Phoridae (Berjano *et al*., 2009; Hipólito *et al*., 2012). *Aristolochia* flowers have been postulated to mimic oviposition sites for flies and generally exhibit a sapromyiophilous or micromyiophilous pollination system mediated through flower scent (Vogel, 1978; Faegri & van der Pijl, 1979; Johnson & Jürgens, 2010). Other studies discuss mimicry of sex pheromones, as trapped flies were either males (Hall & Brown, 1993; Rulik *et al*., 2008) or females (Trujillo & Sérsic, 2006). The flowers of several tropical species emit unpleasant, carrion-like odors (e.g. *A. grandiflora*; Burgess *et al*., 2004). In other species, sweet or slightly unpleasant odors such as decaying fruits, decomposing plants, or fungi were reported (Daumann, 1971; Vogel, 1978; Bänziger & Disney, 2006; Trujillo & Sérsic, 2006). So far, floral scent composition has only been analyzed in a few tropical species. *Aristolochia gigantea* emits carrion-like (dimethyl disulfide), sweet (linalool), and citronella-like (citral, beta-citronellol) odors (Raguso, 2006; Hipólito *et al*., 2012). *Aristolochia arborea* emits fungal odors (mono- and sesquiterpenoids) (Kaiser, 2006a), and *Aristolochia cymbifera* emits a wide array of components from different classes, with dimethyl disulfide and benzyl alcohol being most abundant (Johnson & Jürgens, 2010). Although some data on the scent composition of a few *Aristolochia* species are available, individual components that specifically attract pollinators have not yet been uncovered for any species.

Here, a complete description of the pollination system of the Mediterranean *Aristolochia rotunda* L. is presented. *A. rotunda* is visited by flies belonging to several families (Bibionidae, Cecidomyiidae, Ceratopogonidae, Chloropidae, Sciaridae; Berjano *et al*., 2009). However, it is unknown whether reported visitors of *A. rotunda* flowers carry pollen and truly act as pollinators. Mediterranean *Aristolochia* species produce scents that are hardly detectable for the human nose, and no detailed studies on scent composition are available.

As it is generally assumed that *Aristolochia* mimics brood sites of fly pollinators, and as members of fly families that visit *A. rotunda* have saprophagous, mycetophagous or coprophagous larvae, we tested the hypothesis that *A. rotunda* uses olfactory floral cues to mimic brood sites of its pollinators. Alternatively, flowers may mimic sex pheromones of the pollinators. We identified the pollinators of *A. rotunda*; analyzed the chemical composition of floral scent by dynamic headspace and GC-MS; identified which components are perceived by the pollinators using electroantennographic measurements; and identified behavior-mediating components in field bioassays. All lines of evidence are proof of the existence of a new pollination system in flowering plants.

## Materials and Methods

### Habitat and study sites

*Aristolochia rotunda* L. is distributed in an area ranging across northern Morocco, Spain, throughout the northern Mediterranean, and the Balkans to Turkey and colonizes damp grassy areas and boulders (Nardi, 1984, 1991). The study was performed in the northwestern part of Croatia. Voucher specimens of the plants are deposited at Herbarium Dresdense (DR).

### Pollinators and identification of Diptera

Female-stage flowers were collected and examined for trapped insects during annual field trips from 2009 to 2012. As *A. rotunda* flowers are proterogynous, all collected insects from female-stage flowers that carried *Aristolochia* pollen must have visited an *Aristolochia* flower in the male stage before and are regarded as pollinators (Rulik *et al*., 2008; the most conservative approach). All pollinators were identified to family level (Oosterbroek, 2006) and within Chloropidae individual species were identified (Collin, 1946; Narchuk *et al*., 1970, 1989; Dely-Draskovits, 1981; Beshovski, 1985; Nartshuk & Andersson, 2013). The same approach was applied to flies attracted in biotests (see the section ‘Bioassays’). From pollen-carrying individuals, *c*. 50% were randomly selected to determine the pollen load by scanning electron microscopy (Supra 40 VP SEM; Carl Zeiss, Oberkochen, Germany). *Aristolochia* pollen was found on all inspected individuals. As only *A. rotunda* occurs at the study sites and other *Aristolochia* species that might potentially be present are pollinated by Phoridae (Rulik *et al*., 2008), the pollen must belong to *A. rotunda*. The collected fly specimens were deposited at Senckenberg Naturhistorische Sammlungen, Museum für Tierkunde Dresden (MTD).

### Scent sampling

#### *Aristolochia rotunda* flower scent

The volatiles emitted from *A. rotunda* flowers (*n* = 7), were collected by dynamic headspace methods (Dötterl *et al*., 2005b) in the field. Preliminary analyses of glasshouse plants revealed an unpredictably variable emission of volatiles by single flowers (e.g. the total amount of hexyl butyrate and (*E*)-2-hexenyl butyrate emitted ranged from 0 to 81 ng min^–1^ per flower), and the amount may not exceed the detection threshold. Therefore, 24–50 female-stage flowers from eight to 20 plants were sampled using flame-treated, cold forceps and pooled in an oven bag (8 × 15 cm; Toppits®, Minden, Germany). The effect of cutting was found to be minor, as scent collected *in situ* from two cultivated plants yielded the same components. However, aliphatic hydrocarbons occurred in small amounts in samples collected from attached flowers, but in high amounts in samples collected from detached flowers in the field, suggesting that the concentrations of aliphatic hydrocarbons increased as a result of cutting.

Flower volatiles were collected in adsorbent tubes containing a 1 : 1 (v/v) mixture of Tenax-TA (mesh 60–80) and Carbotrap B (mesh 20–40) (both Supelco, Bellefonte, PA, USA). Two approaches were used. In the first, volatiles were collected for 60–75 min from two samples using a glass pipette (length 7 cm) containing 30 mg of the adsorbent mixture. The mixture was fixed in the tubes using glass wool (Dötterl *et al*., 2009). Subsequently the adsorbed components were eluted with 100 μl (24 female-stage flowers) and 200 μl (29 female-stage flowers) high-grade acetone (Merck, Darmstadt, Germany). In the second approach, flowers from the other five samples were put into a plastic bag for 80–100 min, from which volatiles were collected for 3 min in small tubes (ChromatoProbe quartz microvials; Varian Inc., Palo Alto, CA, USA; length 15 mm, inner diameter 2 mm) filled with 3 mg of the adsorbent mixture. In all cases, the air containing volatiles was sucked (flow rate = 200 ml min^−1^) through the adsorbent tubes by a membrane pump (G12/01 EB; Rietschle Thomas Inc., Puchheim, Germany). Acetone samples and the small loaded adsorbent tubes were stored at 4°C during fieldwork and at −25°C in the laboratory before GC-MS (all samples) and GC-electroantennographic detection (GC-EAD; an acetone-scent sample) analyses. To unambiguously assign components to floral scent, leaf volatiles (*n *= 4; five to 28 pooled leaves) and ambient control samples were collected and analyzed for comparison.

#### Heteroptera scent

*Aristolochia rotunda* flower scent analyses revealed chemicals previously identified in true bugs of the family Miridae (Zhang & Aldrich, 2004). This preliminary finding was substantiated by a literature search on the semiochemicals described from Miridae (http://pherobase.com; see Supporting Information, Table S1) to check for an overlap with components derived from *A. rotunda*. Additionally, we analyzed semiochemicals of true bugs collected at the study sites.

True bugs release large amounts of volatiles from scent glands located in the thorax upon predator attack (e.g. praying mantis, spiders, ants) (Byers, 2006). These volatiles are detected by flies to find an appropriate food source (Eisner *et al*., 1991; Aldrich & Barros, 1995; Zhang & Aldrich, 2004) that is eaten by a predator (kleptoparasites; sensu Sivinski *et al*., 1999; Iyengar, 2008). Among those flies that feed on secretions of mirid bugs are Chloropidae (Zhang & Aldrich, 2004), which have been identified as pollinators in the present study. To simulate a predator attack, living individuals of Miridae and Lygaeidae (one to three samples per species; one to three individuals per sample; see Tables1, S2) were put in a small oven bag and squeezed from the outside with a forceps. After 20 min, volatiles were collected for 3 min using the small adsorbent tubes and the setup described earlier. Samples collected from empty oven bags served as negative controls. Heteroptera were collected in the field and identified as described in the section ‘Bioassays’.

**Table 1:** *Aristolochia rotunda* components eliciting antennal responses in *Trachysiphonella ruficeps* and their presence in freshly killed Heteroptera (Miridae, Lygaeidae)

Total amount of scent trapped per flower per true bug (ng (20 min)^–1^)		Miridae		Lygaeidae	
	Aristolochia rotunda*	*Capsus ater*	*Notostira elongata*	*Peritrechus gracilicornis*	*Xanthochilus quadratus*
	(*n *= 7)	(*n* = 3)	(*n* = 1)	(*n* = 2)	(*n* = 1)
	Median (min–max)	Min–max	177.7	Min–max	918.4
	153.6 (113.1–282.4)	281.1–759.5		214.4–228.2	
Aliphatic esters					
Hexyl isobutyrate**	+	+	−	−	−
(*Z*)-3-Hexenyl butyrate**	+	+	+	−	−
(*E*)-3-Hexenyl butyrate	+	+	−	−	−
Hexyl butyrate	++++	++++	+	+	+
(*E*)-2-Hexenyl butyrate	++++	+++	+++	+	+
Hexyl 2-methylbutyrate	+++	−	−	−	−
Heptyl butyrate**	+	+	−	−	−
Hexyl hexanoate	+++	+	+	−	−
(*E*)-2-Hexenyl hexanoate	+++	+	++++	−	−
Octyl butyrate	++	+	−	−	−
Decyl acetate	+	−	−	−	−
(*E*)-2-Hexenyl (*E*)-2-hexenoate	+	−	−	−	−
Decyl butyrate	+	−	−	−	−
Aliphatic alcohols					
(*Z*)-3-Hexen-1-ol + (*E*)-2-Hexen-1-ol + hexanol	++	−	−	−	−
Aliphatic hydrocarbons					
Undecane	++++	−	−	−	−
Tridecane	++++	−	−	−	−
Pentadecane	++++	−	−	−	−
Unknowns (seven substances pooled)	+++	−	−	−	−

Sample size (*n*), median, minimum (min), and maximum (max) total absolute amounts of scent trapped. Relative amounts of single components are provided as: −, not detected; +, ≤ 0.5%; ++, 0.6–1.0%; +++, 1.1–5.0%; ++++, > 5.1%. Within a component class, substances are ordered based on retention time on a ZB-5 column. A list of all components found in *A. rotunda* and the heteropterans is available in Table S2.

* Relative amounts are based on all seven samples, while absolute amounts are based only on five thereof (see Table S2).

** *T. ruficeps* responded to a synthetic sample only.

### GC-MS

To identify the volatiles of *A. rotunda* and *Heteroptera* spp., headspace samples were analyzed on a Varian Saturn 2000 mass spectrometer coupled to a Varian 3800 gas chromatograph equipped with a 1079 injector (Varian Inc.), which had been fitted with the ChromatoProbe kit (Dötterl & Jürgens, 2005). To analyze the acetone scent samples, 1 μl of each sample was placed in a quartz vial, which was then injected in the GC by means of the ChromatoProbe (Dötterl *et al*., 2005a). The scent-loaded small traps were directly inserted into the injector by means of the ChromatoProbe and analyzed by thermal desorption. For all samples, the injector split vent was opened and the injector was heated to 40°C to flush any air from the system. The split vent was closed (acetone-scent samples) or set to 10 (scent-loaded small traps) after 2 min. The injector was heated at 200°C min^−1^, then held at 200°C for 4.2 min, after which the split vent was opened and the injector cooled down. Separations were achieved with a fused silica column ZB-5 (5% phenyl polysiloxane; 60 m long, inner diameter 0.25 mm, film thickness 0.25 μm; Phenomenex, Torrance, CA, USA). Electronic flow control was used to maintain a constant helium carrier gas flow of 1.0 ml min^−1^. The GC oven temperature was held for 7 min at 40°C, and then increased by 6°C min^−1^ to 250°C and held for 1 min. The MS interface worked at 260°C and the ion trap at 175°C. Mass spectra were taken at 70 eV (in EI mode) with a scanning speed of 1 scan s^−1^ from *m/z* 30 to 350. GC-MS data were processed using the Saturn Software package 5.2.1. Individual components were identified based on the NIST 08, Wiley 7, and Adams (Adams, 2007) mass spectral databases or the database provided in MassFinder 3, and confirmed by comparison of retention times with published data (Adams, 2007). Structural assignment of some components was confirmed by comparison of both mass spectrum and GC retention time with authentic standards. We estimated total scent emission by injecting known amounts of monoterpenoids, benzenoids, and fatty acid derivatives (added to small tubes). The mean response of these components (mean peak area) was used to determine the total amount of each component extracted from the small tubes (for more details, see Dötterl *et al*., 2005b).

### Determination of biologically active components

#### Electrophysiological analyses

The components from *A. rotunda* flowers that were perceived by four females of *Trachysiphonella ruficeps* (Macquart, 1835) (= *T. pygmaea* (Meigen, 1838)), the most abundant pollinator, were identified by GC-EAD according to Dötterl *et al*. (2005a). Based on these results, a synthetic mixture containing several EAD-active volatiles was produced to confirm their activity in three different female individuals of *T. ruficeps*. The flies were collected in the field from female-stage flowers, sent to Bayreuth by express mail, and used for measurements within 3 d after collection. Glass micropipettes filled with insect ringer solution (8.0 g l^−1^ NaCl, 0.4 g l^−1^ KCl, 4 g l^−1^ CaCl_2_) and connected to silver wires were used as electrodes for EAD. After excising the head of a fly, the reference electrode contacted the occiput (where the head was cut off the thorax) while the recording electrode contacted the tip of the third segment of one of the antennae. The GC-EAD system used consisted of a gas chromatograph (Vega 6000 Series 2; Carlo Erba, Rodano, Italy) equipped with a flame ionization detector (FID) and an EAD setup (heated transfer line, two-channel USB acquisition controller) provided by Syntech (Hilversum, the Netherlands). A volume of 1 μl of an acetone-flower scent sample was injected splitless at 60°C, followed by opening the split vent after 1 min and heating the oven at a rate of 10°C min^−1^ to 200°C. The final temperature was held for 5 min. A ZB-5 column was used for the analyses (length 30 m, inner diameter 0.32 mm, film thickness 0.25 μm; Phenomenex). The column was split at the end by the four-arm flow splitter GRAPHPACK 3D/2 (Gerstel, Mülheim, Germany) into two deactivated capillaries (length 50 cm, inner diameter 0.32 mm) leading to the FID and EAD setup. Makeup gas (He, 16 ml min^−1^) was introduced through the fourth arm of the splitter.

#### Bioassays

**Figure 1:**
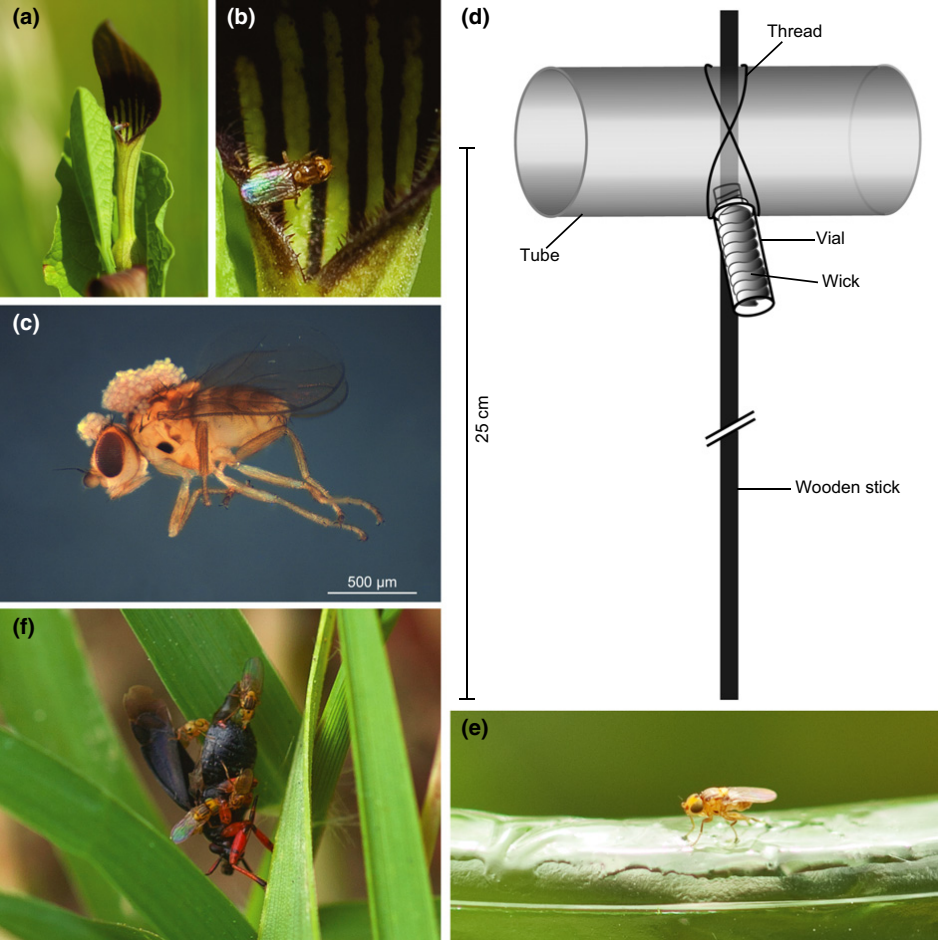
Actors involved in the newly discovered mimicry system and bioassay setup. (a) Flower of *Aristolochia rotunda* with a *Trachysiphonella ruficeps* individual at the margin of the flower tube. (b) Magnification of panel (a). (c) *T. ruficeps* pollinator collected from an *A. rotunda* flower in the female stage, carrying *Aristolochia* pollen on the head and thorax. (d) Flytrap used for bioassays. (e) *T. ruficeps* pollinator sticking to insect glue of a trap loaded with Mix ‘Aristolochia-Miridae’. (f) *T. ruficeps* individuals on a freshly killed *Capsus ater*. The fly in the upper part of the picture is feeding on *C. ater* secretions.

Assays were performed in natural populations of *A. rotunda* to test whether the pollinators are attracted to: (1) either individual components or a mixture of floral volatiles; (2) a synthetic mixture containing only EAD-active components that occur in both *A. rotunda* (present study) and Miridae (based on http://pherobase.com; see the section ‘Heteroptera scent’); and (3) freshly killed mirid bugs, as outlined in the following:

1. To test whether the flower scent of *A. rotunda* attracts pollinators, a synthetic mixture (‘Aristolochia’ mix) was prepared using 75 μl hexyl butyrate, 75 μl (*E*)-2-hexenyl butyrate, 10 μl butyl butyrate, 10 μl pentyl butyrate, 10 μl hexyl isobutyrate, 10 μl heptyl butyrate, 10 μl octyl butyrate, 10 μl undecane (10^−2^ in acetone, v/v), 10 μl tridecane (10^−2^ in acetone, v/v), and 10 μl pentadecane (10^−2^ in acetone, v/v). For details on the origin and purity of the components used, see Table S3. To obtain a sample for bioassays, the mixture was diluted in acetone to a final concentration of 10^−2^ (v/v). The composition contained all commercially available EAD-active components as well as butyl butyrate and pentyl butyrate, both of which elicited antennal responses in a sciarid fly, collected from an *A. rotunda* flower in a glasshouse in Bayreuth. Behavioral experiments were performed with sticky traps (Fig.1d). A trap consisted of a 50 ml Falcon tube (VWR, Darmstadt, Germany) from which the lid and the bottom were removed. The tube was fixed to a wooden stick (length 30 cm) after drilling a hole (8 mm diameter) into the tube wall in the middle of the falcon. The tube was covered inside by colorless insect glue (Temmen GmbH, Hattersheim, Germany). For two-choice tests, two flytraps were positioned at a distance of 50 cm apart and at 20–25 cm above ground level in the field. A 2 ml vial (Supelco) was equipped with a 2-cm-long wick (6 mm diameter, for oil lamps; Alschu GmbH, Westheim, Germany; cleaned with acetone). A quantity of the synthetic mix (500 μl) was applied to the wick of one vial, whereas acetone was applied to a second vial as a negative control. The vials were fitted into the hole of the falcon tube so that the volatiles were released into the tube. Tests were performed at the end of May 2011. Starting in the morning, traps were exposed for 12–57 h. All insects that were attracted to the traps and stuck to the glue were collected and identified.
  To compare the attractiveness of the ‘Aristolochia’ mix with individual components thereof and an additional negative control, multiple-choice tests (four replicates) were performed using the setup described earlier. Nine traps per replicate were arranged in a row at a distance of 50 cm from each other. The baits consisted of 500 μl of a 10^−2^ dilution (in acetone, v/v) each of the ‘Aristolochia’ mix, (*E*)-2-hexenyl butyrate, hexyl butyrate, octyl butyrate, heptyl butyrate, pentyl butyrate, butyl butyrate, as well as hexyl isobutyrate. Pure acetone served as a negative control. The tests were performed in May 2011 and lasted for 11–46 h beginning in the morning.
2. In May 2012, bioassays were performed using the synthetic mix ‘Aristolochia-Miridae’. The semiquantitative composition of components in the mixture resembled that of the flower volatiles as determined by dynamic headspace-GC-MS: 42% hexyl butyrate, 38% (*E*)-2-hexenyl butyrate, 9% hexyl hexoate, 7% (*E*)-2-hexenyl hexanoate, 3% octyl butyrate, 1% (*Z*)-3-hexenyl butyrate, and 0.03% heptyl butyrate. The mixture was prepared by mixing (in this order) 150 μl hexyl hexanoate, 90 μl (*E*)-2-hexenyl hexanoate, 50 μl octyl butyrate, 100 μl (*E*)-2-hexenyl butyrate, 140 μl hexyl butyrate, 0.8 μl (*Z*)-3-hexenyl butyrate, and 0.2 μl heptyl butyrate. Bioassays were performed using a jar (8 cm diameter, 9 cm high) equipped with insect glue (Temmen GmbH) on the upper portion of the inner glass wall (*c*. 1 cm) and the upper edge. The jar was placed at ground level in the grass. Two 2 ml vials were prepared as described earlier: one was loaded with 150–200 μl of the ‘Aristolochia-Miridae’ mix (10^−2^ in acetone, v/v), and the other with an equal amount of acetone. The vials were not offered to the flies simultaneously, but one after the other in the same jar. At first, the acetone vial was placed in the middle of the jar for a test period of 10 min. No insect was attracted to this negative control. Subsequently, the acetone sample was removed, and the synthetic mixture was placed in the glass. Another 10 min later, the flies that were attracted and stuck to the glue (Fig.1e) were removed. The absolute amount of the ‘Aristolochia-Miridae’ mix emitted during the biotest (144 ng (10 min)^–1^ using 150 μl of the mixture) is equal to the amount emitted by a single plant with two to 19 female-stage flowers (62–584 ng (10 min)^–1^; B. Oelschlägel & S. Dötterl, unpublished data) as well as by one squeezed individual of Miridae (34.3–185 ng (10 min)^–1^) (Table S4).
  Although the synthetic mixture and the acetone control were not offered simultaneously, this experiment was treated statistically as a two-choice assay (13 replicates over a distance of at least 20 m). All tests were performed at midday and in the afternoon when flies were found to be active.
3. The attractiveness of different heteropteran species to pollinators was tested in May 2012. Bioassays were performed at midday and during the afternoon hours. An 8 × 8 cm piece of grayish-brown cardboard was covered with insect glue and placed *c*. 30 cm above the grass. No insect responded to this cardboard alone. To simulate a predator attack, a true bug was squeezed with flame-treated, cold forceps and immediately positioned in the middle of the cardboard. After 5–45 min, all insects that remained on the cardboard were collected. The heteropteran test individuals were collected from the population either by hand or using a scoop net.
  Subsequently to bioassays, the bugs used in the tests were identified in the laboratory (Wagner, 1970; Zaitzeva, 1977; Moulet, 1995; Péricart, 1998a,b,c; Derzhansky & Péricart, 2005): Miridae – *Capsus ater* (Linnaeus, 1758) (*n* = 8), *Notostira elongata* (Geoffroy, 1785) (*n* = 7); Lygaeidae – *Xanthochilus quadratus* (Fabricius, 1798) (*n* = 2), *Peritrechus gracilicornis* (Puton, 1877) (*n* = 4); Pentatomidae – *Aelia acuminata* (Linnaeus, 1758) (*n* = 1); and Rhopalidae – *Myrmus miriformis (*Fallén, 1807) (*n* = 3).

### Statistics

The significance of the bioassay results were tested using either the exact binomial test of goodness-of-fit (for two-choice bioassays) or the randomization test of goodness-of-fit (10 000 replicates; for multiple-choice tests) (McDonald, 2009). For both tests, the null hypothesis that all employed baits were equally attractive to the pollinators was tested. The sex ratio of chloropids collected from flowers of *A. rotunda* and attracted to the ‘Aristolochia-Miridae’ mix was compared using a 2 × 2 table *χ*^2^-test (STATISTICA 12, 2012; StatSoft (Europe) GmbH, Hamburg, Germany).

## Results

### Pollinators of *A. rotunda*

Only 268 (32%) out of 845 arthropods trapped inside 1860 female-stage flowers carried pollen and were regarded as pollinators. By far the largest proportion (88%) belonged to the dipteran family Chloropidae, while the remaining were unidentified Ceratopogonidae. Three chloropid species were identified: *Trachysiphonella ruficeps* (208 ♀, 17 ♂, Fig.1a–c), *Oscinimorpha minutissima* (Strobl, 1900) (8 ♀), and *Aphanotrigonum femorellum* (Collin, 1946) (2 ♀).

### Flower scent analysis and biological activity of scent components in *T. ruficeps*

Dynamic headspace GC-MS analyses revealed a complex flower scent comprising a plethora of components. With the exception of two terpenoids, exclusively aliphatic components were detected (Table S2), among which aliphatic esters with 49 different components were the most diverse class. The most abundant component classes were aliphatic hydrocarbons and aliphatic esters, comprising 72 and 25% of the flower scent, respectively.

**Figure 2:**
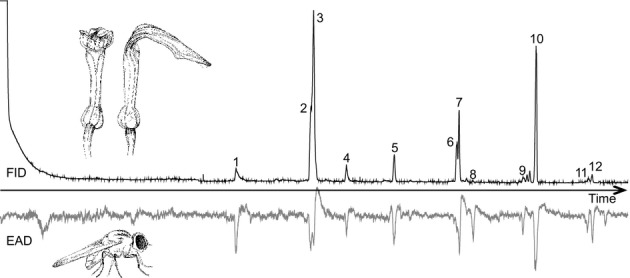
*Aristolochia rotunda* flower scent components are physiologically active in *Trachysiphonella ruficeps*. The black track represents the flower scent chromatogram (FID) of *A. rotunda*, and the gray track the respective electroantennogram (EAD) of *T. ruficeps*. Twenty-six components (see Table1) consistently elicited a response in the antennae of female flies used for the measurements, of which the ones that elicited the strongest responses are indicated: 1, undecane; 2, hexyl butyrate; 3, (*E*)-2-hexenyl butyrate; 4, hexyl 2-methylbutyrate; 5, tridecane; 6, hexyl hexanoate; 7, (*E*)-2-hexenyl hexanoate + octyl butyrate; 8, decyl acetate; 9, unknown aliphatic hydrocarbon; 10, pentadecane; 11, unknown aliphatic; 12, decyl butyrate.

Twenty-six responses to flower volatiles were obtained from *T. ruficeps* in GC-EAD measurements (Table1; Fig.2). The most variable biological active component classes were aliphatic esters (13 EAD-active substances), with hexyl butyrate and (*E*)-2-hexenyl butyrate being the most abundant substances.

**Figure 3:**
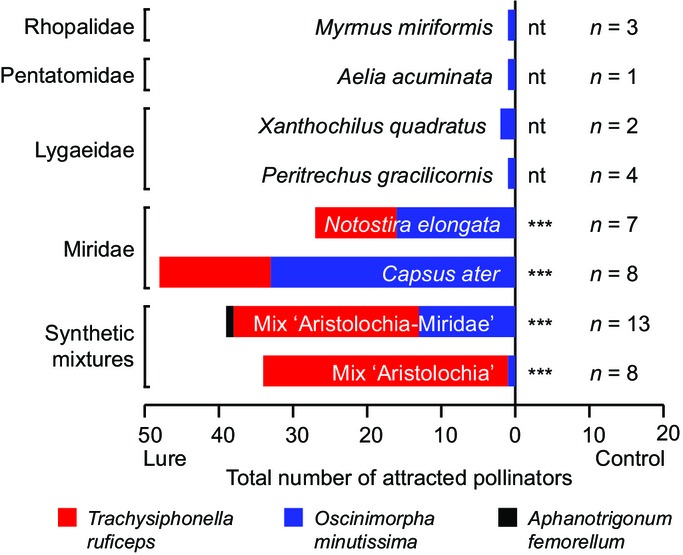
Behavioral response of pollinators to synthetic volatile mixtures and freshly killed true bugs. Two-choice bioassays (*n*, number of replicates) performed in the field testing a bait (synthetic mixtures or freshly killed true bugs) against a negative control (acetone for synthetic scent mixtures and the blank trapping devices for heteropterans). In all bioassays, there was no response to a negative control. The main pollinator of *Aristolochia rotunda*,*Trachysiphonella ruficeps*, only responded to synthetic mixtures and Miridae spp. but not to individuals of the other heteropteran families. The sex ratios of the flies are given in Tables S5 and S6. Exact binomial tests were performed (scent vs control) when applicable: ***, *P* ≤ 0.001; nt, not tested, owing to the small number of attracted flies.

According to behavioral assays, the EAD-active components also elicited a behavioral response. The synthetic mixture of EAD-active substances (‘Aristolochia’ mix: main aliphatic esters and hydrocarbons) attracted 33 *T. ruficeps* (30 females, three of undetermined sex) and a single female of *O. minutissima* (Fig.3; Table S5). Using a multiple-choice assay, individual esters responsible for the attractiveness of the mixture were identified (Table S6). The attractiveness of the individual components, the mixture, and the control were significantly different (randomization test; *χ*^2^ = 48, *P* < 0.001). The synthetic mixture as well as the individual substances, hexyl butyrate, (*E*)-2-hexenyl butyrate, and heptyl butyrate, each attracted female *T. ruficeps* without significant differences in attractiveness (randomization test; *χ*^2^ = 4.7, *P* = 0.21). The control and the four other esters never attracted pollinators.

### Co-occurrence of individual components in *A. rotunda* flowers and mirid bug volatiles and their biological activity

Pherobase contains 51 substances reported for mired bugs (Table S1), of which 17 were also identified among *A. rotunda* flower volatiles (Table S2). Seven of the latter elicited an electrophysiological response in electroantennographic analyses (hexyl butyrate, (*E*)-2-hexenyl butyrate, hexyl hexoate, (*E*)-2-hexenyl hexanoate, octyl butyrate, (*Z*)-3-hexenyl butyrate, heptyl butyrate). The synthetic ‘Aristolochia-Miridae’ mix containing these seven compounds was highly attractive to chloropid pollinators (Fig.1e), resulting in attraction and trapping of individuals of all three identified chloropid species (Fig.3). As already seen in flies collected from flowers, the attractiveness of the synthetic mixture was again biased towards females (*χ*^2^-test, sex ratio flowers vs sex ratio bioassay: *χ*_(df = 1)_^2^ = 2.9, *P* = 0.1) (Table S5).

### Attractiveness of Heteroptera to *A. rotunda* pollinators

Besides Miridae, three other heteropteran families (Lygaeidae, Pentatomidae, and Rhopalidae) were found in high abundance in the vicinity of flowering *A. rotunda*. Consequently, individuals from all four families were used as freshly killed bait. While all provided Heteroptera attracted chloropids within 1–3 min, including *O. minutissima*, only the two mirid species, *Capsus ater* and *Notostira elongata*, attracted *A. rotunda*'s main pollinator *T. ruficeps* (Figs1f, 3; Table S5). Per replicate, at least four times the number of individuals were trapped using mirid bugs instead of other heteropterans. Again, female individuals of *T. ruficeps* and *O. minutissima* were preferentially attracted.

### Analyses of heteropteran volatiles collected at the study site

Chemical analyses of freshly killed heteropteran volatiles collected at the study sites revealed significant differences in qualitative and quantitative composition between mirid and nonmirid species. The two mirid species show a more diverse volatile composition consisting of 19 (*C. ater*) and 16 (*N. elongata*) substances, respectively, whereas the lygaeid mixture is composed of only six components (Table S2). Aliphatic esters are the most diverse component class in mirid species (13 and 10 substances, respectively). Five aliphatic esters found in *A. rotunda* flower scent, which proved to be EAD-active in *T. ruficeps*, were identified from both mirid species, and nine components in at least one of the mirid species (Table1). By contrast, only hexyl butyrate and (*E*)-2-hexenyl butyrate were detected in lygaeid species as well, both in much smaller amounts than in mirid species (Table S4).

## Discussion

### Pollinators

Chloropidae as well as Ceratopogonidae were identified as pollinators of *A. rotunda* in this study. Chloropidae, however, have been the most abundant. While members of both families were known to visit *A. rotunda* flowers (Berjano *et al*., 2009), this study provides the first proof of pollen transfer between male- and female-stage flowers. Three chloropid species (almost exclusively females) were identified and one of them, *T. ruficeps*, accounted for 96% of the chloropid pollinators trapped inside the flowers. This species is regarded as the most important pollinator of *A. rotunda* at our study sites. Chloropids occur as flower visitors in several other tropical as well as extratropical *Aristolochia* spp. Although only identified as a pollinator in *Aristolochia arcuata* Masters (Berjano *et al*., 2009), Chloropidae might potentially be important pollinators for many *Aristolochia* spp.

In general, chloropids have rarely been identified as pollinators of angiosperms, but are described as almost exclusive pollinators (*Tricimba* sp.) of *Pleurothallis* orchids, whose flowers strongly smell of fish. The flies regularly lay eggs on the flowers (Borba & Semir, 2001), suggesting that the orchids mimic food sources for the larvae. *Ceropegia* is another genus from which chloropids are described as pollinators (Ollerton *et al*., 2009). Similar to *Aristolochia* (see the next section), however, oviposition has not been observed (Ollerton *et al*., 2009).

### Floral scent and pollinator attraction in *A. rotunda*

Our data provide the first evidence of pollinator attraction mediated by flower scent in *Aristolochia*. Many volatiles released by flowers of *A. rotunda*, among them those that attract pollinators, are also components of the floral scent of other angiosperm flowers (Kaiser, 1993, 2006b, 2011; Knudsen *et al*., 2006). However, these components typically occur only as traces, and it is unknown whether they are relevant as chemical mediators for pollination. More importantly, the behaviorally active aliphatic esters identified here have never been shown to be relevant in floral mimicry of brood sites (Urru *et al*., 2011; Jürgens *et al*., 2013), and the volatile composition found in *A*.* rotunda* is distinct from fetid, brood site-deceptive *Aristolochia* spp. (Johnson & Jürgens, 2010). In addition, we did not observe signs of oviposition in the investigated *A. rotunda* flowers and it has never been proven before that chloropids use Heteroptera for oviposition. As a result of our data, brood-site deception in *A. rotunda* is strongly rejected.

A strong sex-specific bias in pollinators has been observed in other *Aristolochia* spp. as well and resulted in the assumption that sex pheromones serve as attractants (Rulik *et al*., 2008). Flowers of *A. rotunda* attract pollinators from both sexes, although in considerably different numbers. Mimicry of sex pheromones is therefore unlikely.

Instead, the *A. rotunda* scent appears to mimic a food source that is rich in nutrients for egg development or may be used for chemical defense. True bugs from the family Miridae constitute a food source for kleptoparasitic chloropids (Zhang & Aldrich, 2004). In several true bug families, aliphatic esters are released from the metathoracic scent glands (MSGs; Aldrich, 1988), and in Miridae, they are the main components of the emitted volatiles (Knight *et al*., 1984). In Miridae, MSG secretions function as pheromones or defense signals (Kakizak & Sugie, 2001; Staples *et al*., 2002) and are released upon disturbance or predator attack. Two of the secreted components, hexyl butyrate and (*E*)-2-hexenyl butyrate (Byers, 2006), have been shown to be strong attractants for foraging chloropids in North America (*Olcella* sp. and *Conioscinella* sp.) (Zhang & Aldrich, 2004). Chloropids are well-known kleptoparasites that steal food from arthropod predators (e.g. spiders or praying mantids) (Robinson & Robinson, 1977; Aldrich & Barros, 1995; Sivinski *et al*., 1999; Iyengar, 2008). The mirid defense pheromones/allomones were shown to act as kairomones, allowing the chloropids to find looted Heteroptera (Zhang & Aldrich, 2004). Thus, by mimicking the scent of freshly killed true bugs, *A. rotunda* most probably deceives its kleptoparasitic chloropid pollinators. Indeed, this idea is supported by our data on the semiochemicals of true bugs available in the habitat of *A. rotunda*, and the behavioral assays with true bugs (and a synthetic scent mixture thereof). Freshly killed mirid true bugs release several components also found in floral scents of *A. rotunda* (Table1), and a synthetic mixture (‘Aristolochia-Miridae’) containing many of these components turned out to be highly attractive for chloropids. In addition, all three chloropid species identified as pollinators were attracted by the ‘Aristolochia-Miridae’ mix, and the two most abundant pollinators were also attracted when using freshly-killed Miridae as bait (Figs1f, 3). Interestingly, Miridae differ in their semiochemical composition from other families of Heteroptera (the present study and http://www.pherobase.com), and *A. rotunda* seems to imitate not just any Heteroptera, but specifically Miridae species, with *C. ater* and/or *N. elongata* being the likely models.

Ceratopogonidae proved to be less important pollinators of *A. rotunda*. Midges of this family are also known as kleptoparasites and may be deceived in the same or a similar way as the chloropids (Sivinski *et al*., 1999). In 2011, four Ceratopogonidae individuals were attracted by synthetic compounds but not determined to species level. Therefore, it remains unanswered whether the attracted midges belong to the same species as the pollinating Ceratopogonidae. In 2012, no midges of this family were trapped in our bioassays.

### Conclusion

This is the first study that elucidates the interaction between an *Aristolochia* sp. and its pollinators. We unravel a new pollination system, based on flowers that mimic insect semiochemicals to attract kleptoparasitic flies. Flowers mimicking insect semiochemicals to attract pollinators are known from other angiosperms (Schiestl *et al*., 1999; Brodmann *et al*., 2009). However, we demonstrate that *A. rotunda*, and very likely also other deceptive angiosperms, evolved a different pollination strategy, which we would like to describe using the novel term ‘kleptomyiophily’. This pollination system has two novel aspects. First, the plant mimics compounds released from freshly killed insects rather than living insects. Secondly, unlike bees (Brodmann *et al*., 2009), flies deceived by *A. rotunda* are not looking for food for their offspring but for themselves (insect secretions or hemolymph). This pollination system is probably not restricted to *A. rotunda*, as other dipteran candidate pollinators of *Aristolochia* (Cecidomyiidae, Ceratopogonidae, Chloropidae, Empididae, Lonchaeidae, Milichiidae, and Phoridae) (Berjano *et al*., 2009) also show kleptoparasitic behavior (Eisner *et al*., 1991; Sivinski *et al*., 1999). Other genera with trap flowers, such as *Ceropegia* (Apocynaceae), may likewise exhibit this pollination system (Heiduk *et al*., 2010). As this system is highly specific with respect to the pollinators and their food sources mimicked by the flowers, the *Aristolochia* and *Ceropegia* trap flowers probably produce a highly specified array of floral volatiles, which might act as a driver for speciation in these plants.

## Supporting Information

**Table S1**Pherobase survey on chemical substances reported for true bugs of the family Miridae **Table S2** Composition of headspace scent samples **Table S3** Components used as references in scent analyses and for bioassays **Table S4** Amount of EAD-active components **Table S5** Attractiveness of synthetic scent mixtures and Heteroptera to pollinators **Table S6** Attractiveness of synthetic scent mixture and single components thereof to pollinators
