# Supplementary material for: The betrayed thief – the extraordinary strategy of Aristolochia rotunda to deceive its pollinators
Source: New Phytol. 2014 Dec 8;206(1):342–51. doi: 10.1111/nph.13210 (PMC4357391; doi:10.1111/nph.13210)
Supplement: Supplementary file 1 — Table S1Pherobase survey on chemical substances reported for true bugs of the family Miridae Table S2 Composition of headspace scent samples Table S3 Components used as references in scent analyses and for bioassays Table S4 Amount of EAD-active components Table S5 Attractiveness of synthetic scent mixtures and Heteroptera to pollinators Table S6 Attractiveness of synthetic scent mixture and single components thereof to pollinators [file nph0206-0342-sd1.pdf]

New Phytologist **Supporting Information**

The betrayed thief – the extraordinary strategy of *Aristolochia rotunda* to deceive its pollinators

Birgit Oelschlägel, Matthias Nuss, Michael von Tschirnhaus, Claudia Pätzold, Christoph Neinhuis, Stefan Dötterl and Stefan Wanke

Article acceptance date: 6 November 2014

The following supporting information is available for this article:

Table S1 Pherobase survey on chemical substances reported for true bugs of the family Miridae

Table S2 Composition of headspace scent samples

Table S3 Components used as references in scent analyses and for bioassays

Table S4 Amount of EAD-active components

Table S5 Attractiveness of synthetic scent mixtures and Heteroptera to pollinators

Table S6 Attractiveness of synthetic scent mixture and single components thereof to pollinators

Table S1 Pherobase survey on chemical substances reported for true bugs of the family Miridae

**Aliphatic esters**	(*E*)-2-Butenyl butyrate; Butyl acetate; **Butyl butyrate**; Ethyl butyrate; Ethyl hexanoate; Ethyl myristate; Ethyl-2-hexenoate; Heptyl acetate; **Heptyl butyrate***; Heptyl hexanoate; (*E*)-2-Hexenyl acetate; (*Z*)-3-Hexenyl acetate; **(*E*)-2-Hexenyl butyrate***; **(*Z*)-3-Hexenyl butyrate***; **(*E*)-2-Hexenyl hexanoate***; (*E*)-2-Hexenyl (*E*)-2-hexenoate; (*E*)-2-Hexenyl pentanoate; **Hexyl acetate**; **Hexyl (*E*)-2-butenoate**; **Hexyl butyrate***; **Hexyl hexanoate***; **Hexyl (*E*)-2-hexenoate**; **Hexyl propionate**; **(*E*)-2-Octenyl acetate**; (*Z*)-3-Octenyl acetate; (*E*)-2-Octenyl butyrate; (*E*)-2-Octenyl hexanoate; Octyl acetate; **Octyl butyrate***; Octyl hexanoate; **Pentyl butyrate**; Pentyl hexanoate
**Aliphatic alcohols**	**1-Hexanol**; **(*E*)-2-Hexen-1-ol**; **(*Z*)-3-Hexen-1-ol**; (*E*)-2-Octen-1-ol; (*Z*)-3-Octen-1-ol
**Aliphatic aldehydes**	2,4-Dimethyl pentanal; (*E*)-2-Heptenal; (*E*)-2-Hexenal; Nonanal; Octanal; (*E*)-2-Octenal; (*E*)-4-Oxo-2-hexenal
**Aliphatic ketones**	2-Hexanone; 3-Hexanone
**other**	Benzothiazole; γ-Caprolactone; Indole; Methyl salicylate; Phenylacetaldehyde

The table comprises altogether 51 chemical components described from 12 mirid species of 10 genera (pherobase.com, 03/26/2012). Components are sorted alphabetically within their respective substance class. Components co-occurring in *A. rotunda* flower scent (see Table S2) are highlighted in bold, asterisks indicate components being EAD-active in *T. ruficeps*.

Table S2 Composition of headspace scent samples

Component	Retention time (sec)	Content / Comments	*A. rotunda*							Miridae				Lygaeidae		
										C. ater			*N. elongata*	*P. gracilicornis*		*X. quadratus*
			36 ♀ flowers	50 ♀ flowers	25 ♀ flowers	38 ♀ flowers	41 ♀ flowers	24 ♀ flowers*, †	29 ♀ flowers*	1♂	1♂	2♂	1♂	2♀	1♂2♀	1♂
**Aliphatic esters**																
Unknown (MS: 43, 71, 89, 117, 56, 103)	1024		tr	tr	-	-	-	-	-	-	-	-	-	-	-	-
Butyl butyrate	1097	b, l, s	0.2	0.1	0.1	0.2	0.1	0.1	tr	-	-	-	-	-	-	-
(*Z*)-3-Hexen-1-ol acetate	1112	s	-	-	-	-	-	tr	-	-	-	-	-	-	-	-
Hexyl acetate	1124	s	0.7	0.5	0.1	0.7	0.1	0.1	0.1	0.6	0.5	0.6	-	-	-	-
(E)-2-Hexen-1-ol acetate	1129		0.1	0.1	tr	0.3	0.1	0.1	tr	-	-	-	0.5	-	-	-
Butyl (*E*)-2-butenoate	1174		-	-	-	-	-	tr	tr	-	-	-	-	-	-	-
Pentyl butyrate	1254	b, l, s	tr	tr	tr	tr	0.1	tr	tr	tr	0.2	tr	tr	-	-	-
Hexyl propionate	1271		tr	tr	-	-	-	0.0	-	-	-	-	-	-	-	-
(*Z*)-3-Hexenyl isobutyrate	1331		tr	tr	tr	tr	tr	-	-	-	-	-	-	-	-	-
Unknown (MS: 43, 41, 89, 39, 69, 56)	1333		-	-	-	-	-	0.1	-	-	-	-	-	-	-	-
Hexyl isobutyrate	1340	a, s	0.6	0.6	0.2	0.6	1.1	0.3	0.1	tr	tr	tr	-	-	-	-
(*E*)-2-Hexenyl isobutyrate	1341		0.1	0.2	0.1	0.3	0.6	0.1	tr	-	-	-	-	-	-	-
Unknown (MS: 41, 69, 39, 87, 56, 97)	1380		tr	tr	-	-	-	-	-	-	-	-	-	-	-	-
(*Z*)-3-Hexenyl butyrate	1393	a, s	tr	tr	tr	tr	0.1	0.1	tr	tr	tr	tr	tr	-	-	-
(*E*)-3-Hexenyl butyrate	1397	1, l	tr	tr	tr	0.1	0.2	0.2	tr	tr	0.1	0.1	-	-	-	-
Hexyl butyrate	1405	4, a, s	2.7	3.1	0.9	4.4	17.9	13.2	3.1	22.5	45.6	26.6	0.1	0.1	tr	0.1
(*E*)-2-Hexenyl butyrate	1408	4, a, s	0.5	1.5	0.5	1.8	15.2	23.0	1.5	1.9	3.3	1.9	1.9	tr	tr	tr
(*E*)-2-Octenyl acetate + Octyl acetate	1431	s	0.2	0.1	-	-	-	0.1	tr	-	-	-	-	-	-	-
tent. Hexyl (*Z*)-2-butenoate (MS: 69, 86, 39, 41, 87, 56)	1436		-	-	-	-	-	tr	tr	-	-	-	-	-	-	-
(*Z*)-3-Hexenyl valerate / isovalerate (MS: 67, 57, 82, 41, 39, 103)	1465		0.1	0.1	tr	0.1	0.1	tr	tr	-	-	-	-	-	-	-
Hexyl 2-methylbutyrate	1472	2	0.9	0.8	0.1	0.3	5.5	1.8	1.0	-	-	-	-	-	-	-
Unknown (MS: 83, 57, 55, 39, 41, 67,)	1472		1.9	1.6	1.1	2.9	1.1	-	-	-	-	-	-	-	-	-
Hexyl (*E*)-2-butenoate	1478	s	0.2	0.1	tr	0.2	0.1	0.5	0.4	tr	tr	tr	-	-	-	-
(*E*)-2-Hexenyl valerate / isovalerate (MS: 57, 85, 39, 41, 67, 100)	1481		0.1	tr	tr	0.1	0.1	0.1	tr	-	-	-	-	-	-	-
Unknown (MS: 69, 39, 41, 55, 83, 111)	1488		0.1	tr	0.1	0.1	0.2	1.2	0.7	-	-	-	-	-	-	-
Unknown (MS: 67, 57, 82, 41, 123, 97)	1532		tr	tr	-	-	-	-	-	-	-	-	-	-	-	-
Hexyl valerate	1542	s	-	-	-	-	-	0.1	tr	-	-	-	-	-	-	-

Component	Retention time (sec)	Content / Comments								Miridae				Lygaeidae		
			A. rotunda							*C. ater*			*N. elongata*	*P. gracilicornis*		*X. quadratus*
			36 ♀ flowers	50 ♀ flowers	25 ♀ flowers	38 ♀ flowers	41 ♀ flowers	24 ♀ flowers*,†	29 ♀ flowers*	1♂	1♂	2♂	1♂	2♀	1♂2♀	1♂
Heptyl butyrate	1544	a, s	tr	tr	-	-	-	tr	-	tr	tr	tr	-	-	-	-
(*E*)-2-Hexenyl tiglate	1612		tr	tr	tr	tr	tr	tr	tr	-	-	-	-	-	-	-
Unknown (MS: 41, 71, 57, 89, 129)	1619		tr	tr	-	-	-	-	-	-	-	-	-	-	-	-
tent. (Z)-3-Hexenyl hexoate (MS: 67, 82, 41, 55, 99, 117)	1668		tr	tr	tr	tr	tr	0.1	tr	-	-	-	tr	-	-	-
tent. (*E*)-3-Hexenyl hexoate (MS: 67, 82, 43, 55, 99, 117)	1672		tr	tr	tr	tr	0.1	0.1	tr	-	-	-	tr	-	-	-
Geranyl acetate	1673		-	-	tr	-	-	-	-	-	-	-	-	-	-	-
Hexyl hexoate	1677	3, s	1.1	0.7	0.6	2.1	4.1	5.3	1.7	tr	tr	-	0.9	-	-	-
(*E*)-2-Hexenyl hexanoate	1681	1, s	0.7	0.3	0.5	1.6	4.6	6.5	1.2	tr	tr	-	41.7	-	-	-
Octyl butyrate	1682	4, a, s	0.1	0.1	0.1	0.4	1.2	2.2	0.4	tr	tr	-	-	-	-	-
Decyl acetate	1707	2, s	0.3	0.4	0.6	0.2	0.4	0.2	0.6	-	-	-	-	-	-	-
Hexyl (*E*)-2-hexenoate	1747		tr	tr	tr	tr	tr	0.1	tr	-	-	-	-	-	-	-
(*E*)-2-Hexenyl (*E*)-2-hexenoate	1755	1	tr	tr	tr	tr	0.1	0.1	tr	-	-	-	-	-	-	-
Unknown (MS: 71, 43, 114, 143, 89, 99)	1786		tr	tr	-	-	-	0.1	tr	-	-	-	-	-	-	-
Unknown (MS: 69, 43, 87, 103, 129, 55)	1803		0.4	0.1	-	-	-	-	-	-	-	-	-	-	-	-
Undecyl acetate	1832		-	-	0.7	0.1	tr	tr	0.3	-	-	-	-	-	-	-
tent. Decyl isobutyrate (MS: 89, 41, 71, 55, 97, 111)	1875		-	-	-	-	-	-	tr	-	-	-	-	-	-	-
Unknown (MS: 99, 71, 43, 81, 55, 110)	1921		-	-	-	-	-	-	-	-	-	-	1.9	-	-	-
Decyl butyrate	1928	3, s	0.1	0.1	0.1	0.1	0.2	1.1	0.9	-	-	-	-	-	-	-
Hexyl benzoate	1929		-	-	-	-	-	-	-	-	tr	tr	-	-	-	-
Dodecyl acetate	1952	l, s	tr	tr	tr	tr	tr	-	-	-	-	-	-	-	-	-
Unknown (MS: 69, 43, 87, 103, 55, 129)	2008		0.1	tr	tr	tr	0.1	2.0	0.9	-	-	-	-	-	-	-
Unknown (MS: 69, 41, 55, 99, 81, 125)	2013	1	0.2	tr	tr	0.1	0.2	3.3	1.3	-	-	-	-	-	-	-
tent. Dodecyl butyrate (MS: 41, 69/71, 55/57, 97, 89, 83, 111, 125)	2043		tr	tr	tr	tr	tr	tr	0.1	-	-	-	-	-	-	-
**Aliphatic alcohols**																
(*E*)-2-Hexenol	877		-	-	-	-	-	-	-	-	-	-	39.0	-	-	-
(*Z*)-3-Hexen-1-ol + (*E*)-2-Hexen-1-ol + 1-Hexanol	883	1, l, s	0.6	0.3	0.1	0.5	2.2	0.1	0.2	-	-	-	-	-	-	-
1-Hexanol	884	s	-	-	-	-	-	-	-	67.6	48.8	70.5	-	-	-	-
tent. 1-Hexadecanol (MS: 43, 55, 69, 83, 97, 111)	1950		tr	tr	0.1	-	-	-	0.1	-	-	-	-	-	-	-

Component	Retention time (sec)	Content / Comments	*A. rotunda*							Miridae				Lygaeidae		
										C. ater			*N. elongata*	*P. gracilicornis*		*X. quadratus*
			36 ♀ flowers	50 ♀ flowers	25 ♀ flowers	38 ♀ flowers	41 ♀ flowers	24 ♀ flowers*, †	29 ♀ flowers*	1♂	1♂	2♂	1♂	2♀	1♂2♀	1♂
**Aliphatic aldehydes**																
(*E*)-2-Hexenal	859		-	-	-	-	-	-	-	-	-	-	9.0	0.3	0.6	0.7
(*E*)-4-oxo-2-Hexenal	1033		-	-	-	-	-	-	-	tr	0.2	tr	1.1	86.1	82.8	95.6
**Aliphatic acids**																
Butanoic acid	747	s	-	-	-	-	-	-	-	7.1	1.0	-	-	-	-	-
**Aliphatic hydrocarbons**																
Nonane	935	s	1.5	1.2	0.3	0.7	1.1	-	-	-	-	-	-	-	-	-
Decane	1103	s	2.6	2.5	4.2	2.8	6.1	0.1	0.1	-	-	-	-	-	-	-
Undecane	1266	3, s	61.6	67.2	81.2	58.1	27.0	1.5	1.4	-	-	-	-	-	-	-
(*E*)-3-Nonene	1310	l	tr	tr	tr	0.1	0.2	tr	tr	-	-	-	-	-	-	-
Dodecane	1418	l, s	0.7	0.7	1.7	0.7	0.1	tr	0.1	-	-	-	-	-	-	-
a Tridecene (MS: 41, 55, 69, 83, 97, 111)	1552		0.1	tr	tr	tr	0.1	-	-	-	-	-	-	-	-	-
Tridecane	1560	2, l, s	14.3	13.5	2.8	15.9	4.3	2.2	8.5	-	-	-	-	-	-	-
Tetradecane	1696	l, s	0.3	0.1	0.1	0.1	0.2	0.3	0.9	-	-	-	-	-	-	-
tent. Pentadecadiene (MS: 67, 81, 95, 39, 55, 109)	1797		tr	tr	-	-	-	-	-	-	-	-	-	-	-	-
a Pentadecene (MS: 55, 69, 41, 83, 97, 111)	1802	3, l	0.1	0.1	tr	0.1	0.2	0.7	1.0	-	-	-	-	-	-	-
Pentadecane	1823	4, s	5.2	2.7	3.0	3.2	3.0	23.9	64.7	-	-	-	-	-	-	-
Heptadecene (MS: 67, 81, 95, 39, 55, 109)	2027	2	tr	tr	tr	tr	tr	1.7	2.4	-	-	-	-	-	-	-
Heptadecadiene or Heptadecine (MS: 55, 41, 67, 79, 93, 108)	2033	2	0.1	tr	tr	tr	tr	0.8	0.7	-	-	-	-	-	-	-
Heptadecene (MS: 41, 55, 69, 97, 83, 111)	2035	2	tr	tr	tr	tr	0.1	3.1	2.8	-	-	-	-	-	-	-
**Unknown aliphatics**																
Unknown (MS: 67, 39, 41, 55, 82, 97)	864		-	-	-	-	-	-	-	0.3	0.2	0.2	-	-	-	-
Unknown (MS: 56, 41, 69, 85, 105, 120)	985		0.2	0.2	tr	0.3	1.2	tr	tr	-	-	-	-	-	-	-
Unknown (MS: 55, 112, 83, 97, 39, 67/69)	1058		-	-	-	-	-	-	-	-	-	-	tr	2.1	3.9	tr
Unknown (MS: 83, 55/57, 41, 71, 113, 95/97)	1170		-	-	-	-	-	-	-	-	-	-	1.2	11.5	12.8	3.7
Unknown (MS: 57, 67, 39, 83, 100, 127)	1275		-	tr	tr	tr	0.1	-	-	-	-	-	-	-	-	-
Unknown (MS: 55, 41, 69, 83, 97, 111)	1287		tr	tr	-	-	-	-	-	-	-	-	-	-	-	-

Component	Retention time (sec)	Content / Comments	***A. rotunda***							**Miridae**				**Lygaeidae**		
										C. ater			***N. elongata***	***P. gracilicornis***		***X. quadratus***
			36 ♀ flowers	50 ♀ flowers	25 ♀ flowers	38 ♀ flowers	41 ♀ flowers	24 ♀ flowers*, †	29 ♀ flowers*	1♂	1♂	2♂	1♂	2♀	1♂2♀	1♂
Unknown (MS: 67, 39, 81, 55, 95, 110)	1304		tr	tr	tr	tr	0.1	tr	tr	-	-	-	-	-	-	-
Unknown (MS: 43, 99, 71, 55, 87, 115)	1337		-	-	-	-	-	-	-	-	-	-	2.1	-	-	-
Unknown (MS: 69, 39, 55, 82, 97, 111)	1442		-	-	-	-	-	0.1	tr	-	-	-	-	-	-	-
Unknown (MS: 55, 41, 69, 83, 97, 109)	1461		-	-	-	-	-	tr	-	-	-	-	-	-	-	-
Unknown (MS: 81, 71, 43, 55, 96, 125)	1483		-	-	-	-	-	-	-	tr	tr	tr	-	-	-	-
Unknown (MS: 69, 39, 79, 55, 99, 87)	1492		-	-	tr	tr	tr	0.1	-	-	-	-	-	-	-	-
Unknown (MS: 70, 41, 89, 55, 98, 129)	1506		-	-	-	-	-	-	-	tr	tr	tr	-	-	-	-
Unknowns (multiple substances, MS: 41, 56, 69, 85, 103, 129)	1543		0.1	0.1	tr	0.1	0.2	0.1	tr	-	-	-	-	-	-	-
Unknown (MS: 55, 41, 67, 85, 97, 111)	1548		0.1	0.1	0.1	0.1	0.1	-	0.1	-	-	-	-	-	-	-
Unknown (MS: 41, 55, 67/69, 79, 97, 111)	1553		-	-	-	-	-	0.1	0.1	-	-	-	-	-	-	-
Unknown (MS: 55, 41, 69, 83, 97, 111)	1602		tr	0.1	tr	tr	tr	0.1	0.1	-	-	-	-	-	-	-
Unknown (MS: 43, 55, 83, 67, 101, 127)	1616		tr	tr	-	-	-	-	-	-	-	-	-	-	-	-
Unknown (MS: 55, 41, 69, 97, 83, 111)	1660		tr	tr	tr	-	-	-	tr	-	-	-	-	-	-	-
Unknown (MS: 67, 43, 81, 54, 95, 110)	1665		tr	tr	-	-	-	tr	tr	-	-	-	-	-	-	-
Unknown (2 substances, MS: 55, 41, 69, 83, 97, 111)	1686	l	tr	tr	tr	tr	tr	-	0.1	-	-	-	-	-	-	-
Unknown (MS: 55, 41, 69, 83, 97, 111)	1735		tr	tr	0.1	tr	tr	tr	0.1	-	-	-	-	-	-	-
Unknown (MS: 57, 41, 67, 82, 103, 157)	1740		tr	tr	-	-	-	tr	tr	-	-	-	-	-	-	-
Unknown (MS: 69, 43, 87, 103, 56, 159)	1781		tr	tr	tr	tr	tr	0.1	tr	-	-	-	-	-	-	-
Unknown (MS: 41, 55, 69, 83, 97, 111)	1810		0.4	0.1	-	-	-	1.4	0.7	-	-	-	-	-	-	-
Unknown (MS: 41, 55, 69, 83, 97, 111)	1862		-	tr	-	-	-	-	0.1	-	-	-	-	-	-	-
Unknown (MS: 67, 81, 39/43, 54, 96, 110)	1907		-	-	-	-	-	0.1	-	-	-	-	-	-	-	-
Unknown (MS: 69, 41/43, 57, 87, 103, 127)	1909		tr	tr	-	-	-	0.1	0.1	-	-	-	-	-	-	-
Unknown (MS: 69, 57, 39, 81, 99, 110)	1915		tr	tr	-	-	-	0.1	-	-	-	-	-	-	-	-
Unknown (MS: 67, 79/81, 55, 41, 95, 123)	1919	2	-	-	-	-	-	0.1	tr	-	-	-	-	-	-	-
Unknown (2 substances, MS: 55, 41, 69, 83, 97, 111)	1922		0.1	0.1	-	-	-	1.0	0.6	-	-	-	-	-	-	-
Unknown (MS: 67, 43, 81, 55, 95, 110)	1933		-	-	-	-	-	0.1	tr	-	-	-	-	-	-	-
Unknown (MS: 41, 55, 97, 67, 81, 111)	2038		-	-	-	-	-	-	tr	-	-	-	-	-	-	-
Unknown (MS: 41, 69, 109, 55, 95, 79/81)	2069		-	-	-	-	-	-	tr	-	-	-	-	-	-	-
Unknown (MS: 67, 81, 95, 41, 55, 109)	2083		-	-	-	-	-	tr	tr	-	-	-	-	-	-	-
Unknown (MS: 67, 81, 95, 39/41, 55, 109)	2088		-	-	-	-	-	tr	tr	-	-	-	-	-	-	-
Unknown (MS: 41, 55, 69, 83, 97, 111)	2095		-	-	-	-	-	tr	tr	-	-	-	-	-	-	-

Component	Retention time (sec)	Content / Comments	A. rotunda							Miridae				Lygaeidae		
										C. ater			N. elongata	P. gracilicornis		X. quadratus
			36 ♀ flowers	50 ♀ flowers	25 ♀ flowers	38 ♀ flowers	41 ♀ flowers	24 ♀ flowers*, †	29 ♀ flowers*	1♂	1♂	2♂	1♂	2♀	1♂2♀	1♂
Unknown (MS: 67, 95, 55, 41, 79, 107)	2096		-	-	-	-	-	tr	-	-	-	-	-	-	-	-
Unknown (MS: 79, 93,67, 41, 55, 107)	2119		-	-	-	-	-	tr	-	-	-	-	-	-	-	-
Unknown (MS: 69, 81, 41, 95, 109, 55)	2152		-	-	-	-	-	0.1	0.2	-	-	-	-	-	-	-
**Terpenoids**																
Terpenoid (MS: 67, 81, 95, 43, 123, 55)	1634	l	tr	tr	tr	tr	tr	-	tr	-	-	-	-	-	-	-
(*E*)-ß-Caryophyllene	1753	l, s	tr	tr	-	-	-	0.1	-	-	-	-	-	-	-	-
**Total absolute amount (ng) per flower/true bug per 20 min:**			**113.1**	**153.6**	**282.4**	**175.3**	**142.5**	**n.a.**	**n.a.**	**281.1**	**759.5**	**702.4**	**177.7**	**214.4**	**228.2**	**918.4**

Relative and absolute amounts of components of the flower scent of *A. rotunda* and volatiles trapped from freshly killed Heteroptera (Miridae: *Capsus ater*, *Notostira elongata;* and Lygaeidae: *Peritrechus gracilicornis*, *Xanthochilus quadratus*). Scent components are sorted by substance class and retention time. *, Acetone sample; †, Sample used for EAD measurements; tr, traces (relative amount < 0.05%).

Content/Comments:

1–4, Number of EAD responses in 4 EAD-runs with the natural flower sample.

a, EAD response using synthetic substances.

b, EAD measurement employing synthetic substance elicited no response.

l, small amount in leaf samples.

s, component confirmed by synthetic substance.

Table S3 Components used as references in scent analyses and for bioassays

Substance	Origin	Purity
(*E*)-2-Hexenyl butyrate	SAFC Sigmal Aldrich Chemie GmbH Steinheim, Germany	≥96%
(*E*)-2-Hexenyl hexanoate	TCI Europe N.V., Zwijndrecht, Belgium	>95%
(*Z*)-3-Hexenyl butyrate	SAFC Sigmal Aldrich Chemie GmbH Steinheim, Germany	≥98%
Acetone	Merck, Schwalbach/Ts., Germany	99.8%
Butyl butyrate	SAFC Sigmal Aldrich Chemie GmbH Steinheim, Germany	98%
Decyl acetate	SAFC Sigmal Aldrich Chemie GmbH Steinheim, Germany	≥95%
Decyl butyrate	SAFC Sigmal Aldrich Chemie GmbH Steinheim, Germany	97%
Heptyl butyrate	SAFC Sigmal Aldrich Chemie GmbH Steinheim, Germany	≥98%
Hexyl (*E*)-2-butenoate	SAFC Sigmal Aldrich Chemie GmbH Steinheim, Germany	≥95%
Hexyl butyrate	SAFC Sigmal Aldrich Chemie GmbH Steinheim, Germany	≥98%
Hexyl hexanoate	SAFC Sigmal Aldrich Chemie GmbH Steinheim, Germany	≥97%
Hexyl isobutyrate	SAFC Sigmal Aldrich Chemie GmbH Steinheim, Germany	≥98%
Octyl butyrate	SAFC Sigmal Aldrich Chemie GmbH Steinheim, Germany	≥98%
Pentadecane	Sigma Chemicals CO. St. Louis, MO, USA	99%
Pentyl butyrate	WAKO Pure Chemicals Industries, Ltd.	≥98%
Tridecane	Sigma Chemicals CO. St. Louis, MO, USA	99%
Undecane	Sigma Chemicals CO. St. Louis, MO, USA	99%

Table S4 Amount of EAD-active components

		Miridae		Lygaeidae	
	A. rotunda *n*=5	*C. ater* *n* =3	*N. elongata* *n* =1	*P. gracilicornis* *n* =2	*X. quadratus* *n* =1
Hexyl isobutyrate	0.5–1.6	tr-tr	-	-	-
(*Z*)-3-Hexenyl butyrate	tr-0.1	tr-tr	tr	-	-
(*E*)-3-Hexenyl butyrate	tr-0.3	0.1-0.9	-	-	-
Hexyl butyrate	2.5-25.6	63.2-346.6	0.2	0.1-0.2	0.5
(*E*)-2-Hexenyl butyrate	0.6-21.7	5.3-24.8	3.4	tr-tr	tr
Heptyl butyrate	0-tr	tr-0.1	-	-	-
Hexyl hexoate	1.1-5.9	0-tr	1.5	-	-
(*E*)-2-Hexenyl hexanoate	0.5-6.6	0-tr	74.2	-	-
Octyl butyrate	0.2-1.6	0-tr	-	-	-

Absolute concentration range (in ng per flower per 20 min and ng per individual per 20 min, respectively) in dynamic headspace samples of EAD-active components found of both *A. rotunda* and true bugs.

tr, absolute amount <0.05 ng; -, not detected.

Table S5 Attractiveness of synthetic scent mixtures and Heteroptera to pollinators

Bait	*n*	Total number of trapped pollinators		*P****
		Control	Bait	
**Synthetic mixtures**				
Mix "Aristolochia" *	8	0	34 (30♀ *T. ruficeps*, 3 *T. ruficeps* (unknown sex), 1♀ *O. minutissima*)	<0.001
Mix "Aristolochia-Miridae" **	13	0	39 (2♂23♀ *T. ruficeps*, 4♂9♀ *O. minutissima,* 1♀ *A. femorellum*)	<0.001
**Heteroptera**				
**Miridae**				
C. ater	8	0	48 (1♂14♀ *T. ruficeps*, 9♂24♀ *O. minutissima*)	<0.001
N. elongata	7	0	27 (11♀ *T. ruficeps*, 4♂12♀ *O. minutissima*)	<0.001
**Lygaeidae**				
P. gracilicornis	4	0	1 (♀ *O. minutissima*)	nt
X. quadratus	2	0	2 (1♂1♀ *O. minutissima*)	nt
**Pentatomidae**				
A. acuminata	1	0	1 (♀ *O. minutissima*)	nt
**Rhopalidae**				
M. miriformis	3	0	1 (♀ *O. minutissima*)	nt

Two-choice bioassays using synthetic mixtures of volatiles, freshly killed Heteroptera as lure, and respective negative controls.

*Components: 75 µl hexyl butyrate, 75 µl (*E*)-2-hexenyl butyrate, 10 µl butyl butyrate, 10 µl pentyl butyrate, 10 µl hexyl isobutyrate, 10 µl heptyl butyrate, 10 µl octyl butyrate, 10 µl undecane (10^{-2} in acetone, v/v), 10 µl tridecane (10^{-2} in acetone, v/v), and 10 µl pentadecane (10^{-2} in acetone, v/v). A 1% solution of this mixture was used for bioassays.

**Components: 150 µl hexyl hexanoate, 90 µl (*E*)-2-hexenyl hexanoate, 50 µl octyl butyrate, 100 µl (*E*)-2-hexenyl butyrate, 140 µl hexyl butyrate, 0.8 µl (*Z*)-3-hexenyl butyrate, and 0.2 µl heptyl butyrate. A 1% solution of this mixture was used for bioassays.

nt, a statistical test was not performed due to the small number of attracted flies.

*** *P*-value for exact binomial tests.

n, number of replicates.

Table S6 Attractiveness of synthetic scent mixture and single components thereof to pollinators

Bait	# trapped *T. ruficeps*
Control	0
Mix "Aristolochia"	9
Hexyl butyrate	11
(*E*)-2-Hexenyl butyrate	3
Heptyl butyrate	7
Butyl butyrate	0
Hexyl isobutyrate	0
Octyl butyrate	0
Pentyl butyrate	0

Attractiveness of *A. rotunda* flower scent in a multiple-choice bioassay (*n* = 4) using a synthetic mixture, individual components thereof, and a negative control. All trapped insects were female *T. ruficeps*.
